# Sulphur Rains

**Published:** 1847-12

**Authors:** H. Croft

**Affiliations:** Professor of Chemistry, King’s College, Toronto


*Sulphur Rains.* By H. Croft, Professor of Chemistry, King's College, Toronto.—In the last number of your Journal, I noticed a communication, signed J. H. L., on the subject of the so-called sulphur rains, which have been seen in Toronto during the last few years. Your correspondent makes a very useful remark with regard to the periodical return of the phenomenon; but at the end of the paper he states that the yellow deposit is supposed to consist of pollen, and that further observations are required to confirm this fact.

Perhaps the few following remarks on some of the most important investigations on this subject may not be altogether uninteresting to some of your readers, as explaining a phenomenon so frequently observable in this country.

It was believed in former, and even in comparatively recent times, that the yellow substance often found on water after heavy rains, consisted of flowers of sulphur; but Scheuchzer observed that the substance which fell at Zurich in 1677 and 1653, could not be this body, as on being burnt it did not give off any sulphurous odor. Scheuchzer ascribed the substance to the pollen of the red pine; Elshöltz to that of the Lycopodium clavatum. But Schemeider has shown that it may be derived from a variety of plants; he believes that in March and April it may be ascribed to the alders and hazels; in May and June, to the pines, junipers, and birch; and in July, August, and September, to Lycopodium clavatum, Typha angustifolia, and the different kinds of Equisetum. It has been noticed, that near forests through which a strong wind is blowing, that portion of the land lying in the direction of the wind became covered with the yellow powder. In 1761, there was a heavy fall of it at Bordeaux, covering the ground to the depth of two lines. The members of the Academy of Sciences of Paris convinced themselves that the deposit consisted of the pollen of several species of pine.

A Sulphur rain, (or rather pollen rain) was observed at Copenhagen in 1804. The deposit consisted of the pollen of Lycopodium.

No real sulphur rain has yet been observed, although it might possibly occur in the neighborhood of volcanoes.

To the above extracts, from Kauntz' Meteorology, vol. 3, I would add a few words with regard to the yellow powder which fell in Toronto this summer. Having examined it under a powerful microscope, I convinced myself of its being the pollen of pines. I found that the figures corresponded exactly with Bischoff's plates, representing the pollen of the Pinus strobus (white pine.)—*Bischoff's Terminologie Table* xxxiv.

As is well known, the pollen grains are, in general, simple in form; but it occasionally happens that two or more grow together, and thus produce complicated forms. Such is more particularly the case with pollen grains of the Abietinæ, which consist of a large granule, with two vesicular formations attached to it at each end. These abortive pollen grains may be removed by soaking in oil of turpentine, and then rolling between glass plates; they do not appear to have any contents, but to consist of a simple membrane, covered with a kind of net-work, while the centre granule is perfect. In the early stages of the formation of the pollen, all the granules are equal in size, and of the same structure, and as they increase, one is perfected at the expense of the other two, which, however, remain attached to it.—*Megan's Phlazon Physiologie, book* 3.

The yellow substance, therefore, which was observed at Toronto, consisted of the pollen of the Pinus strobus, (white pine) or Pinus resinosa, (red pine) mixed probably with small granules of the pollen of other plants.—*British Am. Jour.*

Toronto, August 16, 1847.

